# Developing an Online Tool to Promote Safe Sun Behaviors With Young Teenagers as Co-researchers

**DOI:** 10.3389/fdgth.2021.626606

**Published:** 2021-03-23

**Authors:** Rebecca Nguyen, Isabelle M. Clare, Nisali Gamage, Gail A. Alvares, Lucinda J. Black, Prue H. Hart, Robyn M. Lucas, Mark Strickland, Mohinder Jaimangal, James White, Shelley Gorman

**Affiliations:** ^1^Telethon Kids Institute, University of Western Australia, Perth, WA, Australia; ^2^School of Public Health, Curtin University, Bentley, WA, Australia; ^3^National Centre for Epidemiology and Population Health, Research School of Population Health, Australian National University, Canberra, ACT, Australia; ^4^Centre for Ophthalmology and Visual Science, University of Western Australia, Perth, WA, Australia; ^5^Cancer Council Western Australia, Perth, WA, Australia; ^6^Curve Tomorrow, Perth, WA, Australia; ^7^Reach Health Promotion Innovations, Perth, WA, Australia

**Keywords:** sun exposure, skin cancer, vitamin D, online tool, app development, health promotion, co-researchers, teenagers

## Abstract

Despite education about the risks of excessive sun exposure, teenagers in Australia are sun-seeking, with sunburn common in summer. Conversely, some regular (time-limited) exposure to sunlight (that avoids sunburn) is necessary for vitamin D and healthy bones and other molecules important for immune and metabolic health. New interventions are thus required to better support teenagers to make healthy and balanced decisions about their sun behaviors. This paper describes the development of a prototype online tool—a smartphone app—that aimed to foster safe sun practices in teenagers. We recruited young adolescents (aged 12–13 years, *n* = 24) as “co-researchers” to provide ongoing input into the nature and design of the online tool. This age group was selected, as it is a critical time when young people transition from primary education, where “SunSmart” behaviors are entrenched in Australian schools, to high school, where risky behaviors emerge. Through a series of interviews and workshops, we codesigned an Apple iOS smartphone app with the co-researchers, leading health promotion professionals, researchers, and app designers. The developed app, *Sun Safe*, contains educational content relevant to teenagers about safe sun behaviors, complemented by other features requested by co-researchers and stakeholders to help engage young people, including gamified quizzes to test their sun health knowledge, real-time weather data on the UV Index and temperature, a sunscreen application timer, and reminders to check the UV Index. The developed prototype app was rated well by co-researchers, suggesting it is suitable for further feasibility and efficacy testing as an intervention tool to improve knowledge and promote safe sun behaviors by young adolescents.

## Introduction

Sun protection is important for young people in Australia, as intermittent excessive sun exposure (causing sunburn) in childhood and adolescence is a major risk factor for melanoma (1). Indeed, Australian adolescents are twice as likely to experience sunburn on the weekend in summer as adults (~1 in 4 vs. ~1 in 8) (2). As the most common cancer of young adults (15–29-year-olds) in Australia (3), melanoma has significant mortality (1,467 deaths in 2014) (3), morbidity, and health costs ($270M AUD/year) (4). Sun protection messages need to target adolescents (particularly males) who are less likely to engage in the most effective sun protection behaviors and are at increased risk of sunburn (5). However, some sun exposure is necessary to maintain vitamin D for bone health, a range of other biological processes, and disease prevention (6, 7). In Australia, sun exposure is the major source of vitamin D, with only small amounts obtained through dietary sources and supplements. Results from the Australian Health Survey (2011–2012) suggest that young adults are the population at most risk of vitamin D deficiency (blood levels of 25-hydroxyvitamin D of <50 nmol/L) (8), which may be explained by adolescents spending more time indoors as they become young adults (9). Indeed, the prevalence of vitamin D deficiency rises from ~15% in 12–17-year-olds to ~30% in young adults (8). There is thus an ongoing need to improve the sun health knowledge and behaviors of young people, so that they can achieve the right balance of sun protection and sun exposure for optimal health.

While Australian adolescents demonstrate a good level of sun protection knowledge and understand that excessive sun exposure is a primary risk factor for the development of melanoma, they underestimate the prevalence of melanoma and mortality rates in youth and have a poor understanding of the increased risk associated with sunburn in childhood and adolescence (10). Adolescents are also more likely than other age groups to participate in “risky” behaviors around sun protection [reviewed by Koch et al. (9)], which is considered a low priority and unwelcome disruption to their daily activities (9). As young adolescents enter secondary (high) school, support mechanisms for promoting healthy sun behaviors are not as well-established as in primary (elementary) schools (e.g., through the “SunSmart” program of Cancer Council Australia) (11) and coincide with a time of life when risky sun behaviors emerge. The wearing of hats by adolescents may be decreasing, with more choosing to spend time in the shade or indoors as a preferred mode of sun protection (9). These behavioral changes may have unintended consequences, including increased risk for sunburn when in the sun (12), reduced opportunities for (outdoor) activity, and increased risk for vitamin D deficiency.

Based on this evidence, there is a need to continue to build further sun protection programs for the adolescent period to continue to reduce the incidence of melanoma—an almost entirely avoidable cancer—in young people. However, it is not easy to frame health promotion messages that balance both sun safety for protection from sunburn while allowing sufficient sun exposure for optimal synthesis of vitamin D. Indeed, the ultraviolet B wavelengths of sunlight are most important for both sunburn and skin cancer, as well as vitamin D synthesis. Some findings suggest that for most people, optimal vitamin D status is probably best achieved by regular short exposures to sunlight, which are sufficient to maintain or raise circulating levels of 25-hydroxyvitamin D, and insufficient to cause sunburn (13). However, communicating this nuanced message through health promotion activities is challenging, especially when targeted toward young people.

Online tools, specifically those tailored toward young adolescents, could be an engaging and effective means of promoting safe sun practices by young people. Further advantages of online tools include their ease of access, low cost to distribute, and the ability for content and messaging to be rapidly modified as needed. Adolescents, as do most adults, like and use mobile phone and tablet technologies for social networking and gaming particularly on personal devices such as smartphones. Most Australian (~90%) adolescents (aged 14–17) own a smartphone (14), and in general, mobile phone apps are widely used. There is emerging evidence that eHealth-based interventions may reduce risky sun behaviors in adolescents (15, 16), although the effectiveness of these sun health-based apps is not often assessed (17). A program of in-person training complemented by daily SMS text reminders was highly rated by young organ transplant recipients (*n* = 26) and increased their reported use of sunscreen (18). Similarly, increased sunscreen use and reduced tanning behaviors were reported by adolescent participants (*n* = 1,573) 6 months after using a smartphone app (*SunFace*) in which facial-aging technology predicted and visualized the adverse effects of excessive sun exposure on the user's face (19, 20). A 3-month intervention testing the *Solar Cell* app unexpectedly reduced sunscreen use and increased shade use by adult participants (*n* = 604) (21). The *SunSmart* app (developed by Cancer Council Australia) provides real-time information on the UV Index and gives guidance on how to best achieve sun protection depending on the person's skin type, where they are located, and what they are wearing. This app is likely more commonly used by Australian adults and is not necessarily tailored toward younger users.

In this study, we sought to develop a new online tool in consultation with young teenagers as “co-researchers,” leading sun health promotion professionals and researchers, and eHealth developers. We hypothesized that this approach of ongoing consumer engagement during the development of the eHealth tool would increase the likelihood of producing an informative, useful, and engaging tool tailored to the wants and needs of end users (17). Our aim was to develop an online intervention tool that improved sun health knowledge and promoted safe sun practices by young adolescents, which would include effective protection from sunburn and sufficient exposure for vitamin D. An additional goal was to produce a fun and engaging online tool with elements of gamification that could provide intrinsic motivation for use by young people (22). We theorized that a fun, educational online tool developed with adolescent co-researchers would be useful and relevant, accepted by adolescents, and increase better decision-making about protection from excessive sun exposure and exposure for sufficient vitamin D.

## Materials, Equipment, and Methods

### Ethics and Governance

Approval to conduct this study was obtained from the Human Research Ethics Committee of the University of Western Australia (RA/4/20/4424). Informed consent was obtained from both the participants (“co-researchers”) and their parent/guardian.

### Study Location

Perth (Western Australia, WA) is located at a latitude of 31.92°S and longitude of 115.87°E. The UV Index often reaches 12–13 (extreme) in summer and 3–4 (moderate) in winter (23). The average daily hours of sunshine annually are 8 h, increasing to 9–10 h during the months of October through April (24). The average daily mean temperatures in summer and winter are 21–24°C and 12–15°C, respectively (25). Perth encompasses an area of 6,400 kmE^2^ and has an estimated current population of >2 million people (26). It is a coastal city, located ~10 km from the Indian Ocean, adjacent to the Swan River. Due to the long hours of daily sunshine, warm weather throughout much of the year, and proximity to the Indian Ocean and Swan River (Derbarl Yerrigan), those living in Perth have a lifestyle that often involves outdoor activities such as swimming, watersports, walking, and bicycling.

### Stakeholders

Stakeholders included young adolescent co-researchers (12–13-year-olds) from the Perth community, sun safety health promotion staff (from Cancer Council WA), leading Australian academics/researchers (from Australian National University, Telethon Kids Institute, and Curtin University), eHealth technology developers (from Curve Tomorrow, Reach Health Promotion Innovations, and Telethon Kids Institute), consumer and community engagement advocates (Consumer and Community Health Research Network, WA, and Telethon Kids Institute), and officials/reviewers from the Department of Education (WA).

### Participant (“Co-researcher”) Recruitment

Young adolescents were initially recruited *via* parents through a social media strategy. This was conducted on Facebook through the Telethon Kids Institute account that has over 16,000 followers, and included “boosted” (or paid) posts that targeted parents over 30 years of age, as well as sharing with local Facebook groups and “community noticeboards.” Researchers also aimed to recruit participants through an e-mail campaign targeting family, friends, and colleagues who may have had contacts with children interested in participating. Recruitment was completed over a 2-week period (August 2018). All participants were recruited through the study web page, managed using an electronic form developed using Qualtrics software hosted at the University of Western Australia. Eligibility included aged between 12 and 13 years, resided in Perth (WA), and had access to the Internet and an Apple iOS device (i.e., iPhone or iPad). Feedback from participants was obtained *via* telephone interviews and/or through their participation in at least 1 of 3 workshops. These were completed in a 12-month period throughout the development of the tested smartphone app.

### Consumer Recruitment

Two young adolescents were recruited as consumer “research buddies” to be consumer representatives and provide additional ongoing feedback to aid with the development and progress of the study. These young people (one male, one female; aged 12–16 years on recruitment, with informed personal and parental consent) met with researchers throughout the development of the online tool and provided input into the conceptualization of the project and its design and ongoing feedback as the project progressed. These individuals were different from the co-researchers participating in the research project.

### Design Thinking for Online Tool Development

A user-centered design thinking framework based on the Stanford “Design Thinking” five-stage (Empathize, Define, Ideate, Prototype, and Test) methodology (27) was used to develop the online tool. During the Empathize and Define stages, a series of interviews and workshops were conducted with young adolescent co-researchers, with ongoing input from all stakeholders to better understand end user's points-of-view in the context of sun safety behavior. In the following Ideate and Prototype stages, a wireframe (simple mock version) was developed based on the learnings from the previous stages, followed by developing a fully functional online tool (app) in the Test stage that could be downloaded, tested, and iterations made based on feedback. An important aspect of this study design was the ongoing engagement of young people as co-researchers and co-designers in the design, implementation, and evaluation of the wireframe and then fully functional app.

### Co-researcher Interviews: Initial Information Gathering

Ten recruited co-researchers were interviewed through one-on-one semistructured telephone interviews of up to 40 min in length (September 11–21, 2018). The interviews focused on exploring co-researchers' day-to-day activities and how they spent time outdoors and made decisions about their sun health behaviors. This involved discussing what leisure activities participants engaged in on hot summer days, how they considered weather conditions when getting ready to go out each day, and their attitudes toward sun protection and use of technology to help make sun health decisions. Participants were also asked for their input on what they would like from an online tool and how it could be best designed to support their own sun health decision-making. Researchers listened to audio recordings of interviews and categorized content into themes using the online *Trello* platform. Detailed qualitative assessments of these interviews will be reported elsewhere.

### Design Thinking Session (Researchers Only)

A “Design Thinking” session was held with a subgroup of the research team (October 2, 2018) conducting a “How-might-we” brainstorming process that considered requirements and constraints to identify possible ways of developing the wireframe. This process considered the major themes that arose from the interviews, with the specific question: “How might we help young teenagers get sufficient vitamin D from safe sun practices,” with ideas themed and then “sprint questions” developed and assessed by researchers as to whether they could be answered by developing an online tool. Ideas generated during brainstorming were then assigned to parts of an average day, considering how young people use technology to make daily decisions about their sun practices, depending on their planned activities, to determine features that could be included in the wireframe of the tool.

### Workshop 1 With Co-researchers: Brainstorming to Develop an Online Tool

All initially recruited co-researchers were invited to attend this 2-h workshop (October 16, 2018, 4:30–6:30 P.M.). Twenty (*n* = 20) individuals participated. Researchers (“table facilitators”) were seated at tables with co-researcher participants to facilitate discussion, take notes, and make observations. The main goals of this workshop were to identify and prioritize the biggest issues and barriers for healthy sun behaviors in young people and use a “How-might-we” brainstorming to generate ideas for a technology-based solution. The topics for the main discussion were (i) sun protection and harms and (ii) sun exposure benefits. Prompter questions were provided to table facilitators to help initiate and maintain discussion (Supplementary Table 1). A “How-might-we” brainstorming was conducted next, with participants asked to individually write their ideas on Post-it notes, addressing the question: “How might we use technology to support safe sun practices?” Ideas were then rapidly themed, and participants were allowed to vote on their 3 favorite ideas using the online *Mentimeter* platform (www.menti.com) to collate votes *via* secret ballot. An issue that arose on the day of this workshop was that the Wi-Fi at Telethon Kids Institute was not working well, with votes only able to be cast by 13 (of 20) co-researcher attendees. This issue was not experienced at subsequent workshops.

### Wireframe Development

With the knowledge generated from the interviews and Workshop 1 and input from the research team, Curve Tomorrow developed a “wireframe” of the online tool. A wireframe is a basic design of what the online tool may look like. The developed wireframe was hosted on the *InVision* web-based platform (www.invisionapp.com) and contained clickable content that linked features of the wireframe.

### Workshop 2 With Co-researchers: Testing the Wireframe

Eighteen (*n* = 18) co-researchers attended a 2-h workshop (December 6, 2018, 4:30–6:30 P.M.) and were asked to bring or were loaned an iOS device (iPhone or iPad) to test the wireframe. The main goals of this workshop were for co-researchers to assess the developed wireframe tool on an iOS device and determine whether it was appropriate for further development and testing as an online tool. Co-researchers were first asked to think about possible names for the online tool and to present any ideas to researchers facilitating the workshop. Co-researchers were then asked to spend 5 min using each feature of the wireframe, recording their comments on a predesigned worksheet, initially describing their first impressions of the wireframe, and then testing each feature. All feedback was completed anonymously. Co-researchers were asked to vote for their favorite name for the online tool using the *Mentimeter* platform. Co-researchers then rapidly assessed the developed wireframe *via* a poll hosted by *Mentimeter* using a subjective 5-point rating system (Likert scale, from 1 star = strongly disagree to 5 stars = strongly agree) to rate 7 items:

1. I understood the purpose of the app
2. It was easy to find the information I wanted
3. I would download and use this app
4. I would recommend this app to my friends/family
5. This app makes me want to be more sun safe
6. This app has helped me better understand the UV Index
7. The app helps me know more about vitamin D

Co-researchers were also asked an open question during this second poll, which was “What would be the one thing you would include in this app to make it definitely something you would use?” Finally, a “How-might-we” brainstorming session was held to explore a major theme that arose from this question, posing a new question: “How might we use games, increase fun and engagement of the developed wireframe?”

### Researcher Feedback on Wireframe

Across a 7-week period (December 7, 2018–January 25, 2019), researchers provided feedback on the developed wireframe.

### App Development

Feedback collected through testing the wireframe were synthesized and used to inform the development of the app. The fully functional iOS app was developed by Reach Health Promotion Innovations. To develop artwork appealing to intended end users (12–13-year-olds), a competition was conducted *via* the online graphics design platform, 99designs (www.99designs.com.au), asking artists to submit designs for 4 icons to identify four major features on the home page of the app (i.e., “Learn,” “Quizzes,” “Notifications,” “Sunscreen Timer”) and 6 icons to describe six major elements within the “Learn” feature (i.e., “UV Index,” “Harms,” “SunSmart,” “Benefits,” “Vitamin D,” “Sunscreen”) in vector format. Designers were asked to provide artwork suitable for young high school-aged students, which was light-hearted and fun with bright colors and iconography preferably in consistent, unique, and cartoon style. Through this platform, we invited the consumer research buddies (*n* = 2) and young teenagers (*n* = 7) known to the research team to rate the submitted designs. The winning designer was also asked to design an additional 8 weather icons for use on the homepage and in the “View this week” feature and an icon for the app. The developed icons were also used as “stickers” incorporated as a “pack” upon download of the app for use when text messaging on the user's phone. A notification alert sound was purchased from royalty-free music hosted at https://www.pond5.com/royalty-free-music/ for a small fee ($5AUD). Seven short-listed potential notification sounds were independently reviewed and rated by two researchers.

### Beta Testing of App

Through a 3-month period (May 31–August 31, 2019) of beta testing, members of the research team and consumer research buddies tested the developed app for technical flaws in design, reviewed the accuracy of web links and information presented, and provided feedback that could improve the functionality, usability, and esthetics of the app. This included testing the app in locations in Australia (Perth, WA; Albany, WA; Canberra, Australian Capital Territory) and internationally (Barcelona, Spain; Dubai, United Arab Emirates).

### Workshop 3 With Co-researchers: Testing the App

Fifteen (*n* = 15) co-researchers participated in a third 2-h workshop held on June 18, 2019 (4:30–6:30 P.M.). Co-researchers were asked to download the *TestFlight* app from the Australian Apple App Store onto an iOS device. The beta version of the *Sun Safe* app—hosted on *TestFlight*—was then downloaded. Participants were instructed to use the *Sun Safe* app for 20 min before providing qualitative and quantitative feedback. For qualitative feedback, co-researchers completed a worksheet containing questions identical to that of Workshop 2, which required them to record their first impressions and opinions of each feature of the app. Co-researchers then rapidly reassessed the developed app using the same *Mentimeter* poll of Workshop 2, in which a subjective 5-star rating system was used to assess the same seven items as well as the four main “features” of *Sun Safe* (“Weather and UV information,” “Learn,” “Quiz,” and “Sunscreen Timer”).

### Further App Modifications to Enable Pilot Testing

Feedback from researchers (during the beta testing phase) and co-researchers (during Workshop 3) was evaluated by researchers, and further modifications were made to the *Sun Safe* app. The app was also evaluated by the Department of Education (WA) for governance approval to conduct a pilot intervention study in a local school. Approval was originally sought on December 11, 2018, and obtained on December 5, 2019, and included five separate responses to queries raised by internal reviewers at the Department of Education.

### Data Analysis

Demographic data from recruited co-researchers were securely stored in the password-protected *Microsoft Sharepoint* (2013) platform hosted by Telethon Kids Institute. Data were de-identified and analyzed in Excel (Microsoft, v16.38, 2020) and Prism GraphPad [v8.4.3(471), 2020] to obtain the mean and standard deviation (SD). Data from the *Mentimeter* polls were compared using Mann–Whitney tests as data were not normally distributed (determined using Shapiro–Wilk and D'Agostino–Pearson normality tests). Ratings given to each feature of the fully functioning app (collected in Workshop 3) were compared using a Kruskal–Wallis test. For all comparisons, *p* < 0.05 were considered significant.

## Results and Discussion

### Requirements, Constraints, and Considerations of the Online Tool

With initial input from academic researchers working in the Sun Health space and public health advocates from Cancer Council WA, the health promotion message of the online tool was developed, which was for users to “spend some time outdoors being active for vitamin D using sun protection as indicated by the UV Index.” The UV Index is a linear scale (1–11+) that describes the daily danger (from low to extreme) of sunburn due to the intensity of solar UV radiation. The UV Index is directly proportional to the intensity of UV radiation that causes sunburn to human skin. It is widely used by health promotion agencies around the world (including Cancer Council Australia and World Health Organization) as a means to help people make decisions about sun protection. Three specific important and related concepts related to the UV Index were incorporated into educational content, which were that:

- When the UV Index is ≥3 (when you are outside), a combination of sun protection is recommended [including wearing a broad-rimmed hat, covering clothing, sunscreen, seeking shade, and wearing sunglasses; consistent with the “Slip Slop Slap Seek Slide” messaging of the SunSmart campaign of Cancer Council Australia (28)].
- When the UV Index is <3 (when you are outside), sun protection is not recommended. However, if you are outside for extended periods (i.e., the whole 4 h between 10 A.M. and 2 P.M.), sun protection is recommended.
- To maintain a sufficient vitamin D status, teenagers (of all skin types) should spend time outdoors being physically active each day using sun protection as indicated by the UV Index.

Educational content related to sun exposure for vitamin D was also informed by the Position Statement on Sun Exposure and Vitamin D: Risks and Benefits, developed and endorsed by Cancer Council Australia's Principal Health Committee, and other Australian health practitioner and health promotion groups in January 2016 (29).

Young adolescents in Australia already have some knowledge about safe sun exposure habits (10) fostered through their early childhood and primary school education. Underlying educational outcomes necessary to understanding the three concepts outlined above were reiterated and/or introduced in the educational content of the online tool, including definition and factors affecting the UV Index, when sun protection is necessary, dangers of excessive sun exposure in adolescence, how to use sunscreen, what wavelengths of UV radiation cause sunburn and make vitamin D, how some sun exposure is beneficial (for vitamin D and other molecules important for our health), UV radiation and skin types, and alternative (diet) sources of vitamin D. This content was updated and revised by researchers throughout the development of the tool to include new findings from scientific literature.

Further requirements and constraints were as follows:

- The developed tool aimed to complement resources already promoted by the Cancer Council Australia and available online (e.g., SunSmart app).
- Inclusion of gamification elements could provide intrinsic motivation for use by young people (22) and distinguish the developed tool from those that already exist. Active engagement strategies considered included the capacity for social interactions and user-content creation (30).
- As the developed tool was intended for use by young teenagers, it needed to consider parental concerns including safety issues such as exposure to adult sexual content and privacy (30).
- Young people may prefer receiving information about the importance of sun protection through a first-person account from a young melanoma survivor compared to traditional methods (e.g., pamphlet), youth marketing, or social media-based campaigns (10). For this reason, a young person's experience of melanoma was considered for potential inclusion in the online tool.
- We aimed for the developed online tool to have the capacity for use in multiple locations (e.g., at home, school, out and about) and beyond the study location to maximize accessibility and ease of use.

### Consumer “Research Buddy” Input

Two consumer research buddies provided feedback on recruitment and communication with co-researchers, wording of interviews and workshop discussion guidelines and worksheets, when to hold interviews and workshops, and what type of “icebreakers” to use during workshops. Consumer research buddies also reviewed the wireframe and beta version of the app, specifically the wording of content in the “Learn” feature, quiz questions within the “Quiz” feature, and whether target users would likely use the “stickers” developed using the app's artwork/icons. Additional specific feedback is also described below.

### Co-researcher Recruitment

**Figure 1:**
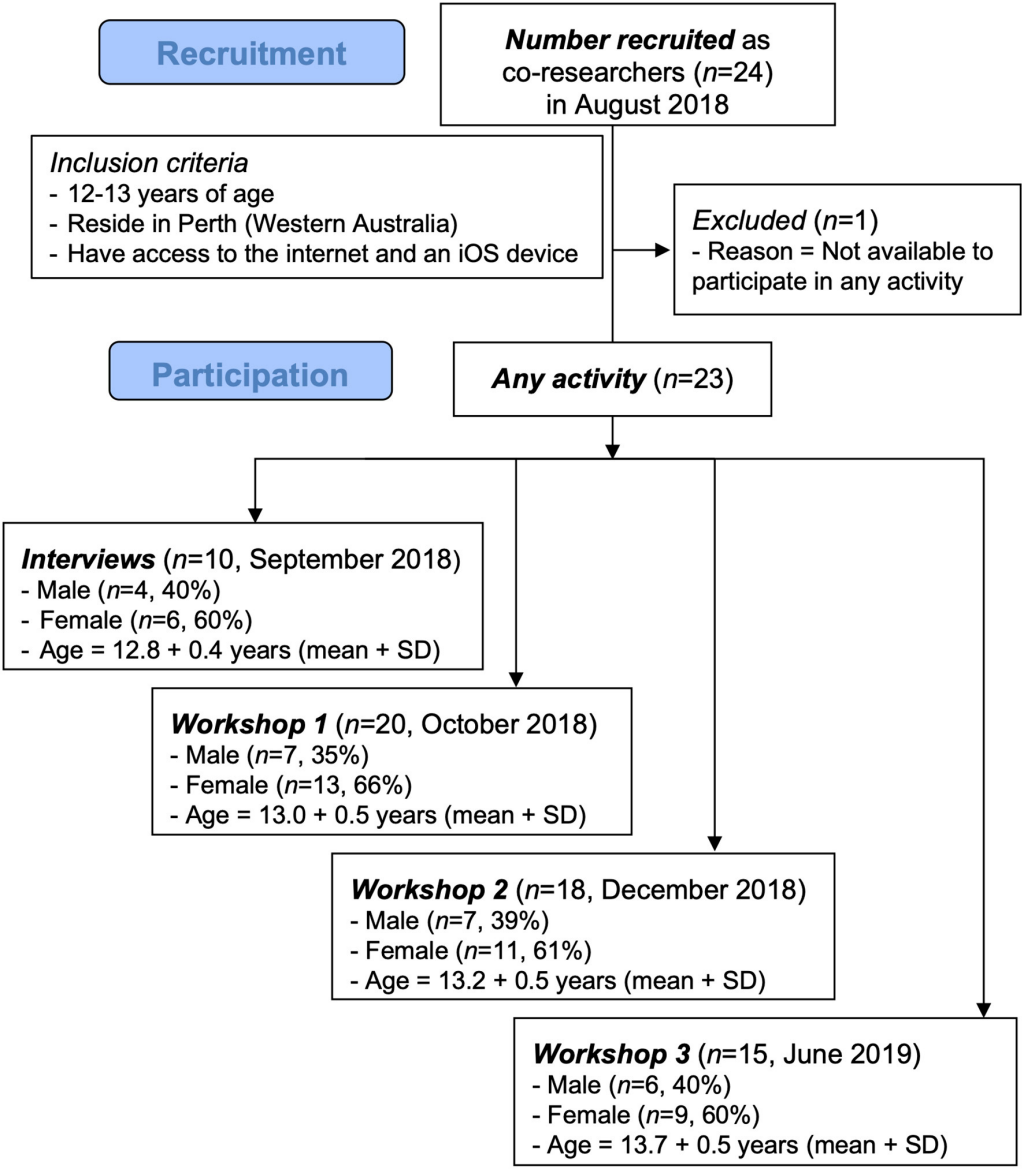
Schema depicting co-researcher recruitment, including number, gender mix, and age for those participating in each activity.

Co-researchers (*n* = 24; seven males, 17 females) were rapidly recruited (within 2 weeks) and were 12.9 + 0.6 (mean + SD) years of age upon recruitment. The number, age, and gender of co-researchers attending each research activity are shown in Figure 1.

### Co-researcher Interviews: Initial Information Gathering

Four themes were identified from interview conversations, in which co-researchers described their behaviors and attitudes toward spending time outdoors and how they made decisions about their sun practices (Supplementary Table 2). A theme of “Major strategies” to help them make decisions about their sun practices centered on participants either finding out what the weather was going to be that day or generally following school guidelines. A second theme of “Challenges” emerged about making these decisions, which were undermined by a lack of understanding by participants of the UV Index, inadequate real-time weather data online that could be readily accessed by co-researchers on the local UV Index, negative attitudes about wearing hats, and neglectful sun practices (by boys in general). Some of these challenges were related to the third theme—“Emotions”—in which participants did not want to be different from their peers but had varying feelings about the personal impacts of excessive sun exposure (ranging from anxiety to dismissal of the personal impacts of minor sunburns). Across these first three themes, a potential role for secondary schools and teachers in perpetuating safe sun practices was observed. Finally, the fourth theme—“Needs”—pointed toward methods through which an online tool could help young people undertake safe sun practices, particularly about providing information on how to effectively use the UV Index and sunscreens and the benefits of vitamin D.

### Design Thinking Session (Researchers Only)

**Figure 2:**
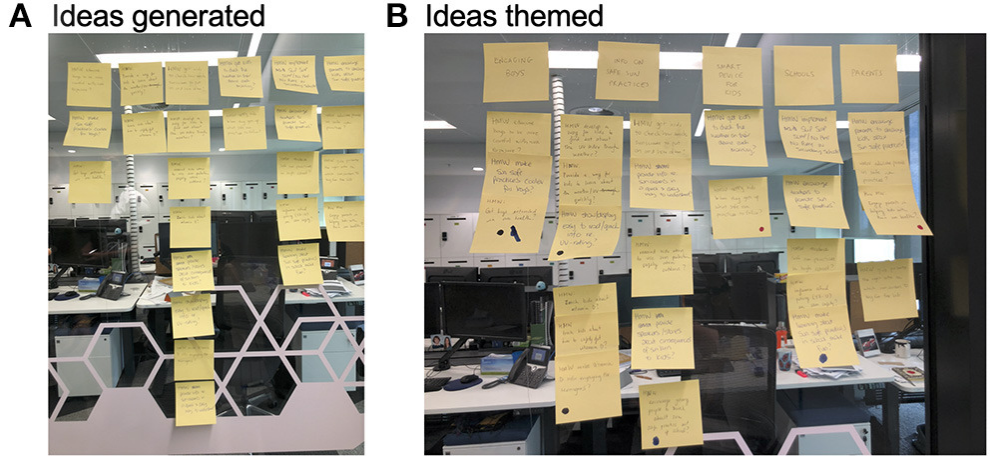
**(A)** Ideas generated by researchers attending a design thinking session from a How-might-we brainstorm addressing the question, “How might we help young teenagers get vitamin D from safe sun practices?” **(B)** These ideas were rapidly categorized into five major themes, which were: (1) Can we engage boys? (2) information on safe sun practices; (3) a smart device for kids; (4) schools; and, (5) parents.

Ideas generated (Figure 2A) by researchers during the brainstorming, addressing the question, “How might we help young teenagers get vitamin D from safe sun practices?,” were rapidly categorized into five major themes (Figure 2B) of “Can we engage boys?,” “Information on safe sun practices,” “Smart device for kids,” “Schools,” and “Parents.” From these themes, six “sprint questions” were developed (Table 1). Three questions were assessed by researchers as being feasibly answered via development of an online tool. These involved creating a (i) go-to resource to educate young people on (ii) vitamin D and (iii) the UV Index. There was less certainty about whether we could make an online tool that specifically engaged males.

**Table 1:** Sprint questions developed in design thinking session.

**Sprint questions developed during the design thinking session**	**Answer^a^**
1. Can we educate kids on vitamin D?	Yes
2. Can we educate kids on the UV Index and weather?	Yes
3. Can we create a “go-to” resource for kids for safe sun practices?	Yes
4. Can we influence school policy on safe sun practices?	No
5. Can we create fashionable safe sun gear?	No
6. Can we engage males to take sun safe practices seriously?	Maybe

a *Researchers assessed the feasibility of answering these questions by developing a new online tool*.

### Workshop 1 With Co-researchers: Brainstorming to Develop an Online Tool

**Figure 3:**
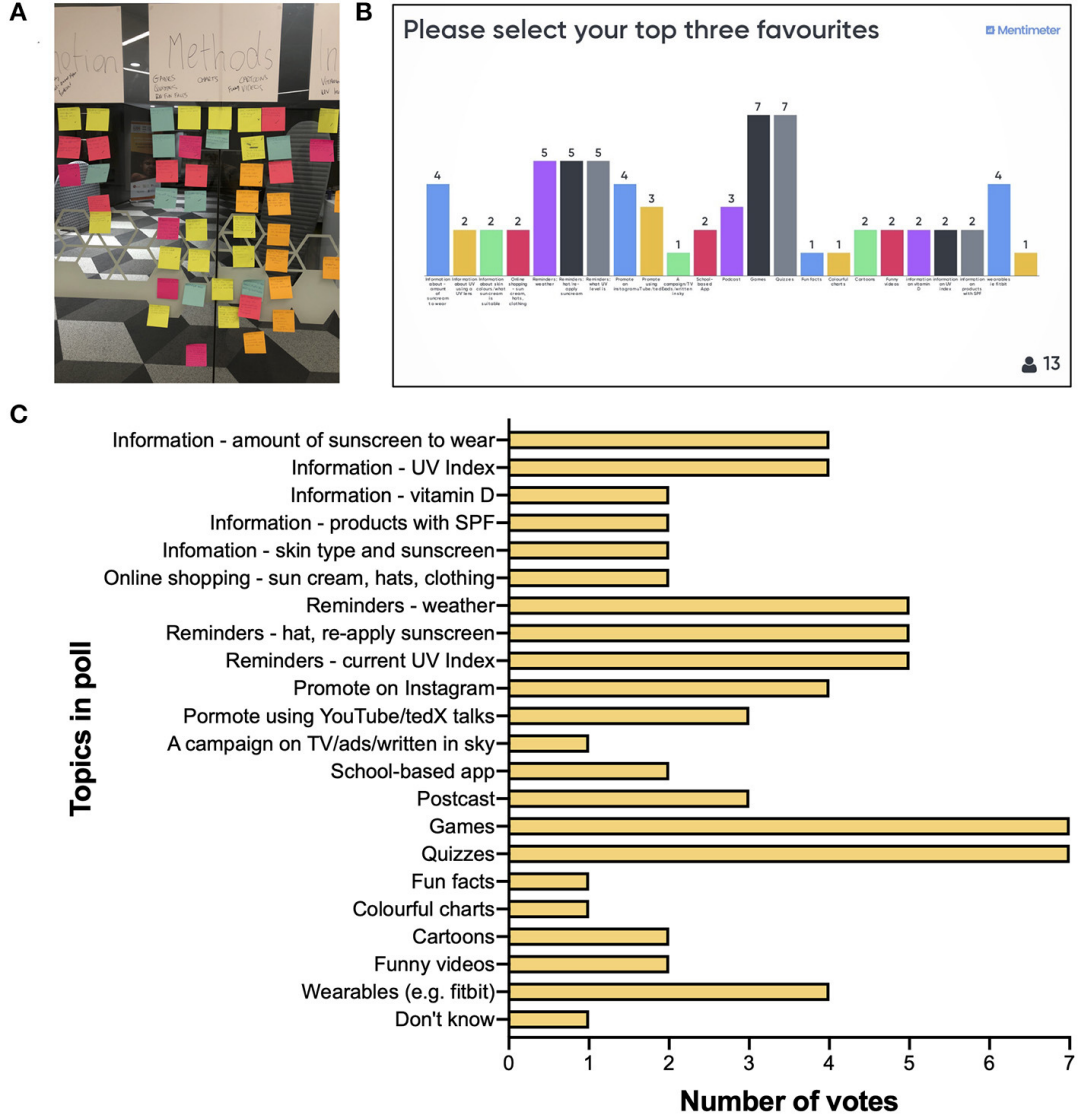
**(A)** Ideas generated by co-researchers (*n* = 20) attending Workshop 1 from a How-might-we brainstorming addressing the question, “How might we use technology to support sun safe practices?” that were categorized into a “Methods” theme (only 37 of 46 final ideas depicted). Co-researchers were invited to vote (*via* secret ballot using the Mentimeter online tool) for their top 3 favorite ideas of 21 selected by researchers at the workshop, with a screenshot of the Mentimeter poll results shown in **(B)** and these results re-graphed (for clarity) in **(C)** for 69 votes cast in total. n.b. Only 13 of 20 co-researchers attending the workshop participated in this poll, during which problems with Wi-Fi were experienced. SPF, sun protective factor.

Major themes that emerged from discussions on (i) sun protection and harms and (ii) sun exposure benefits are summarized in Table 2. These were similar to subthemes that arose through co-researcher interviews (Supplementary Table 2), including a lack of care about sun protection, and that parents had some influence on the co-researcher's sun practices. In general, co-researchers had limited knowledge on potential benefits of sun exposure (Table 2). In the How-might-we (use technology to support sun safe practices) brainstorming, from a total of 80 ideas (Supplementary Table 3), four main themes emerged, including (i) methods to promote safe sun practices (46 ideas; Figure 3A), (ii) means to remind and notify individuals (15 ideas), (iii) information on how to use sunscreens (9 ideas), and (iv) games and quizzes (10 ideas). Across all themes, an app-based idea featured frequently (15 ideas, Supplementary Table 3). Co-researchers were asked to vote for their 3 favorite of 21 discrete/different ideas short-listed by researchers, with 13 co-researchers casting a total of 69 votes (Figure 3B). “Games” and “Quizzes” rated most highly, followed by “Reminders” (Figure 3C). When grouped, most votes (63.8%) were cast for ideas related to “Information” (21.7%), “Reminders” (21.7%), and “Games”/“Quizzes” (20.3%). Co-researcher engagement was assessed by facilitators and communicated through a debriefing session held after the workshop. Facilitators stated that most co-researchers contributed well to discussions, although this varied between attendees, and that prompts prepared in advance (Supplementary Table 1) were useful to initiate and continue discussion. Some co-researchers appeared less enaged than others and were described as “turned off” (n = 1 male co-researcher), “quiet” (n = 2 male co-researchers), or “distracted” (n = 2 female co-researchers); however, facilitators stressed that all individuals contributed, especially during the How-might-we session.

**Table 2:** Discussion themes that emerged during Workshop 1.

**1. Sun protection and harms—When you spend time outdoors with friends and family, how do you get ready to be sun safe?**
• Theme 1: Most participants were not that careful about sun protection • Theme 2: Most participants used sunscreen, but did not reapply it, or use if swimming, or if the weather was cool • Theme 3: Those participants with light-colored skin were more likely to avoid excessive sun exposure • Theme 4: Most participants had experienced a bad sunburn • Theme 5: Most participants knew a person (or a pet) diagnosed with a skin cancer • Theme 6: Most participants received some motivation from parents to be SunSmart
**2. Sun exposure benefits—What have you heard about the benefits of sun exposure?**
• Theme 1: Mixed knowledge of any benefits of sun exposure • Theme 2: A few participants reported that vitamin D was good for bone health • Theme 3: A few participants stated that sun exposure was good for mood and provides warmth
**3. “How-might-we” brainstorming^a^–How might we use technology to support sun safe practices?**
• Theme 1: Methods on how to promote safe sun practices • Theme 2: Means to remind and notify individuals • Theme 3: Information on how to effectively use sunscreens • Theme 4: Games and quizzes

a *Co-researchers were instructed that all ideas were useful contributions and encouraged to “think outside the box,” with a specific listing of all ideas shown in Supplementary Table 3*.

### Wireframe Development

**Figure 4:**
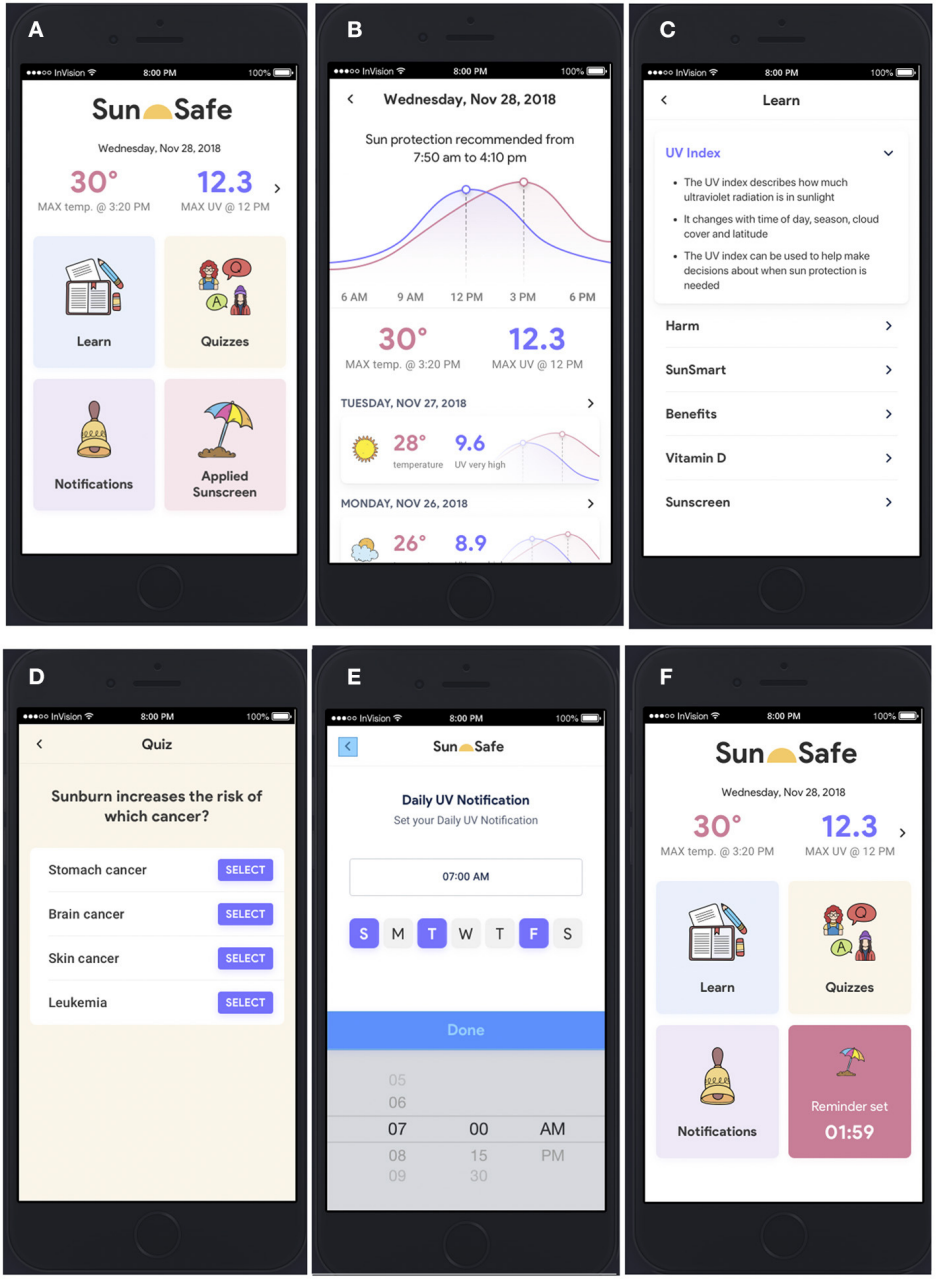
Wireframe of the Sun Safe app. Features of the wireframe included: **(A)** home page with that day's maximal UV Index and temperature data; **(B)** more detailed predictive data on the UV Index and temperature and times when to use sun protection (accessed via arrow on right-hand side of UV Index data on the home page); **(C)** educational content in “Learn” feature; **(D)** embedded quiz content in “Quizzes” feature; **(E)** capacity to set daily “Notifications” to check the UV Index; and **(F)** capacity to set an “Applied Sunscreen” timer (“Reminder set”), which counts down 2 h from when sunscreen is applied and sends a notification to reapply.

Key results from the interviews and Workshop 1 were used to design a wireframe, underpinned by requirements and constraints detailed above. As shown on the home page (Figure 4A) and described in more detail below, the wireframe included the following features: real-time weather data, information on when to use sun protection based on predicted and current UV Index (accessed through a small arrow on the upper right-hand side of the home page, changed later to “View this week”; Figure 4B), educational content (“Learn”; Figure 4C), a quiz to test knowledge of sun health (“Quizzes,” changed later to “Quiz”; Figure 4D), capacity for daily reminders to check that day's UV Index (“Notifications”; Figure 4E), and a timer for sunscreen reapplication (“Applied sunscreen,” changed later to “Sunscreen timer”; Figure 4F). A decision to develop a smartphone app was made due to the frequency at which co-researchers generated app-based ideas in the How-might-we brainstorming of Workshop 1 (Supplementary Table 3) and the capacity for an app to flexibly link together the most highly rated responses generated in this workshop, specifically information, reminders, and games/quizzes (Figure 3C). Other reasons to develop a smartphone app included that most Australian teenagers own a smartphone [>90% (14)], apps can be rapidly updated and deployed, apps are often freely accessible (or cheaply purchased), apps can be specifically designed to enhance end user experiences, and the effects of apps on health knowledge and behaviors can be assessed. The name given to the wireframe/app was *Sun Safe* app.

### Wireframe Content

Weather-based data for the day's maximal UV Index and temperature were shown on the home page (Figure 4A), with more detailed predictive data and sun protection recommendations based on when the UV Index is ≥3 shown in Figure 4B for that day and subsequent days.

The educational content for the “Learn” feature (Figure 4C) was developed based upon that identified as important initially above and through engagement activities with co-researchers. Content was divided into information on the following:

- UV Index—how the UV Index changes with weather conditions and can be used to make decisions about when sun protection is needed;
- Harms—about excessive sun exposure (including sunburn, skin cancer, and eye damage);
- SunSmart—how to use the UV Index and methods for protection from excessive sun exposure;
- Benefits—including vitamin D for bone health;
- Vitamin D—more information about vitamin D, including dietary sources; and
- Sunscreen—how to use sunscreen effectively.

This “Quizzes” feature was included as a method to engage users, which was suggested and highly rated by co-researchers in Workshop 1 (Figure 3). Questions for inclusion in the quiz were developed based upon educational content in the “Learn” feature and informed by the SunSmart online education program hosted at www.generationsunsmart.com.au (Cancer Council WA). While 20 questions were developed, only 5 questions were included and embedded for assessment of the wireframe. These tested the user's knowledge of (i) what cancer was associated with sunburn (Figure 4D), (ii) what wavelengths of UV radiation were responsible for sunburn and making vitamin D, (iii) from what UV Index is sun protection recommended, (iv) that skin exposure is necessary to make vitamin D, and (v) how often sunscreen should be reapplied when needed outdoors.

Co-researchers also requested, and highly rated, the capacity to be notified when to check the UV Index (Figure 3), which was incorporated as the “Notifications” feature (Figure 4E) in which end users could select days and a time of day to be reminded when to check the UV Index. A further means to be reminded of when to next apply sunscreen was included as the “Applied sunscreen” feature in the wireframe. This feature could be tapped to set a 2-h reminder (which counted down) to later send a notification of when to reapply sunscreen (Figure 4F).

### Workshop 2 With Co-researchers: Testing the Wireframe

Most co-researchers attending Workshop 2 used an iPhone (n = 14/18) to test the wireframe, with the remaining using an iPad (n = 2) or did not state the type of device used (n = 2). Most (although not all) first impressions of the wireframe were positive (Table 3). Most co-researchers reviewed the “Quizzes” first (n = 12/18), followed by the “Learn” (n = 9), and then the “Applied sunscreen” (n = 9) features. The main reasons for choosing the “Quizzes” feature were that co-researchers wanted to test their (own) sun health knowledge, that this feature looked interesting, and that they liked playing quizzes because they are fun. This was also reflected in group discussions, in which the favorite feature for two of the three groups was “Quizzes,” with the third group undecided. The same two groups nominated the “Notifications” feature as their least favorite. Most individuals reviewed the “Quizzes” (n = 17/18), “Learn” (n = 16), “Applied sunscreen” (n = 11), and “Notifications” (n = 12) features, with only a few individuals providing feedback on the “View this week” feature (n = 3). One co-researcher did not complete the provided worksheets. Co-researchers were asked to provide feedback on what was missing from the wireframe, with ideas falling into one of eight themes (Table 3), which included expanding content in the “Learn” and “Quizzes” features and providing more ways of engaging the end user (e.g., more variety, colors, pictures, music, and games).

**Table 3:** Feedback on the developed wireframe by co-researchers attending Workshop 2.

**1. First impressions (*****n*** **=** **17)^a^**	
• Very “childish” and not “teenish” and nice colors. I like how you have max temp and time and max UV and the time and date • It's good, it's a little hard to get to, but it runs smoothly like a everyday app	
• I thought it was pretty cool to have an app that lets you know about some of the sun safe stuff	
• The app was cool to look though but it was a bit boring and would have been better if there was some sort of games	
• My first impression of the app was I thought it was really cute, but the colors could be more vibrant	
• Looks cool	
• I like how you can learn a lot and set sunscreen reminders and you can do quizzes and it sends you notifications on the UV ray	
• It would be helpful if it was a real app • Boring, not much there	
• I liked how there were many fun things to do and learn about in one app. The quiz was fun, and I liked the learning section too	
• I like the way you can learn but have fun at the same time. The reminder set I really like and as well with the notifications	
• I thought it looked cool and made me want to try it. The little pictures were also cute. It also looked fun • I think the app looks clean and modern (that's good). It was a little glitchy but it looked helpful	
• I think the app looks really fun, cool, and helpful. The app took a while to load between the pages, but that was probably just the Internet	
• I think it just needs more color, as in more images to get people's attention • I think it looked really good as it was simple and easy to follow	
• I think the app is great and I would definitely use it when I went in the sun. I also recommend to use without Internet as some may not have access to it	
**2. What was missing?**	
**• Theme 1: More variety in general • Theme 2: More colors and pictures • Theme 3: Shopping for sun health products • Theme 4: More quiz questions**	**• Theme 5: More information on cancers • Theme 6: A questions/comments section • Theme 7: Music • Theme 8: Games**
**3. “How-might-we” brainstorm** ^b^ **–“How might we use games, increase fun and engagement of the developed wireframe?”**	
• Theme 1: “Mini-games” and ways to improve the “Quiz” feature of the developed wireframe • Theme 2: Avatars and bitmoji rewards • Theme 3: Music • Theme 4: Voucher rewards • Theme 5: Miscellaneous suggestions	

a *Reported verbatim*.

b *Co-researchers were instructed that all ideas were useful contributions and encouraged to “think outside the box,” with a specific listing of all ideas raised by co-researchers shown in Supplementary Table 4*.

**Figure 5:**
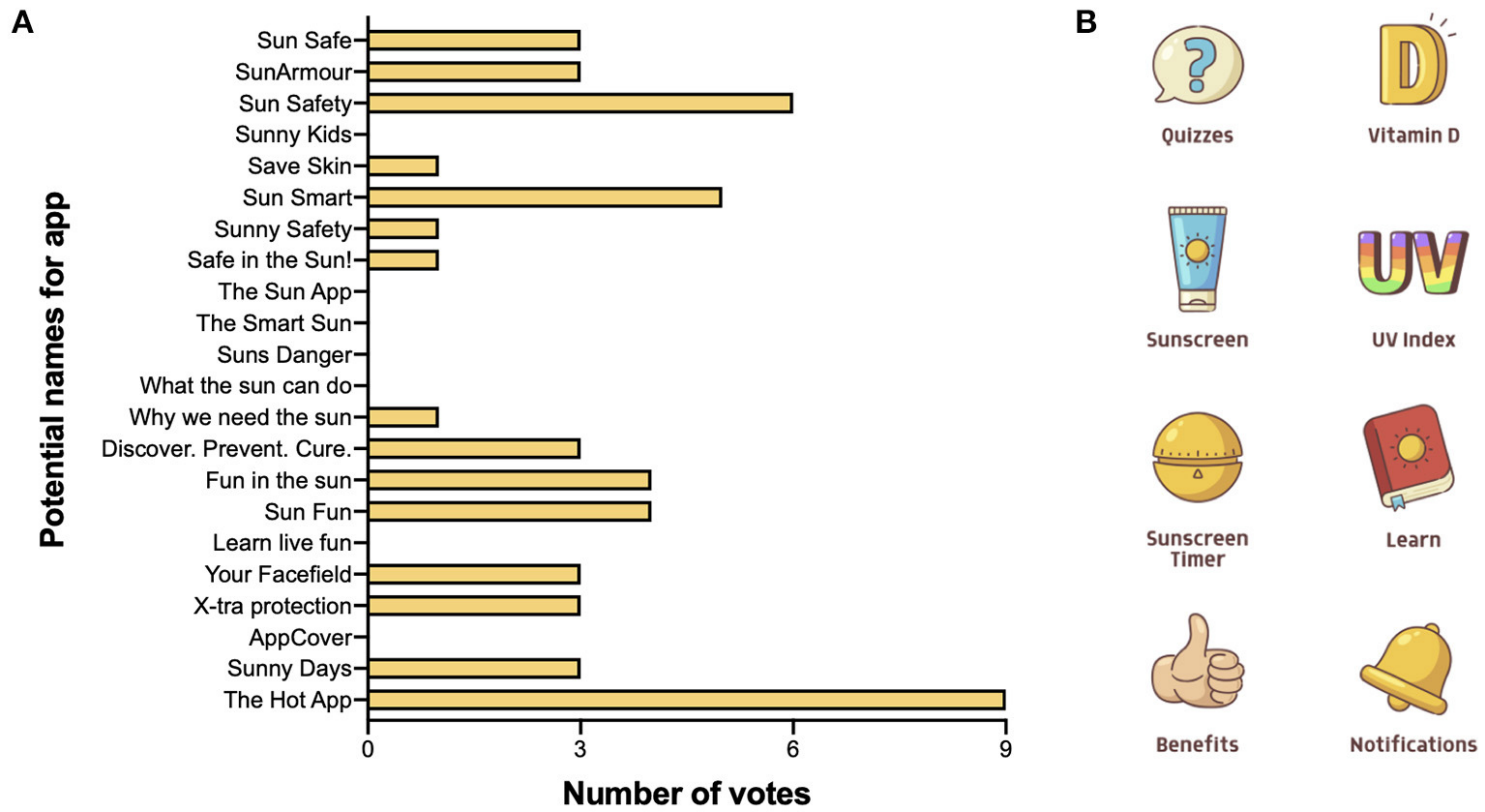
Co-researcher preferred name and artwork for the Sun Safe app. **(A)** A secret ballot was conducted during Workshop 2, asking co-researchers to nomimate their 3 preferred names for the developed wireframe, with 18 co-researchers providing a total of 50 votes. **(B)** App artwork (icons) developed through the 99Designs platform.

**Figure 6:**
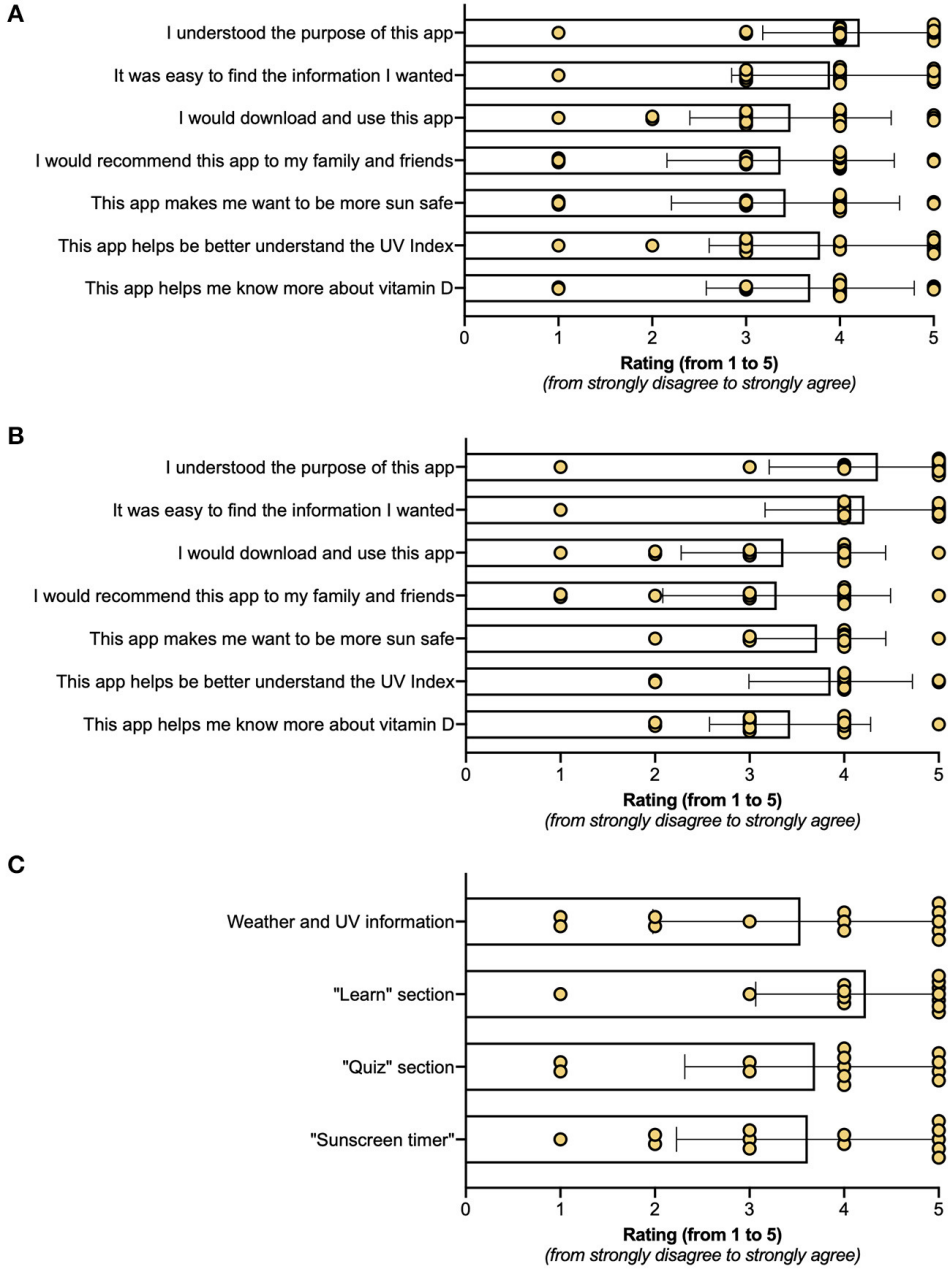
Co-researcher assessments of the wireframe and fully working protype of Sun Safe app. After using the **(A)** wireframe or **(B)** fully developed Sun Safe app for 20 min in Workshops 2 and 3 (respectively), co-researchers were asked to complete a subjective seven-item assessment. In **(A)**, responses from 18 co-researchers (one on 2 occassions), and in **(B)**, responses from 14 co-researchers. In **(C)**, co-researchers attending Workshop 3 were asked to rate four main “features” of the Sun Safe app (responses from 13 co-researchers only). Each item/feature was assessed using a 5-point Likert scale (from 1 star = strongly disagree to 5 stars = strongly agree), with bars denoting mean values and error bars denoting SD, with individual responses also shown.

There were 22 suggested names for the to-be-developed app from co-researchers, with the highest rated names (from 18 co-researchers participating in the online poll of 50 votes) being The Hot App (nine votes), Sun Safety (six votes), and Sun Smart (five votes) (Figure 5A). All attendees (n.b. one co-researcher voted twice) completed the subjective 7-item assessment of the wireframe, which achieved a mean score of 4 or more stars for “I understand the purpose of the app” and was rated by most users with 3 or more stars for other outcomes (Figure 6A). There were 17 responses to an open question that asked co-researchers what to include in the app to make it definitely something they would use, with most responses (58.8%, 10/17) stating this would be a game, followed by real-time data on the UV Index and when to use sun protection (both 23.5%, 4/17). Based on responses to this question, a How-might-we brainstorming session was held to explore the question: “How might we use games, increase fun and engagement of the developed wireframe?” Five major themes emerged in this session (Table 3), which mainly focused on “mini-games” that could be incorporated into the wireframe and suggestions on how to improve the “Quizzes” feature of the developed wireframe (see Supplementary Table 4 for specific responses acquired during this brainstorming session). Most of these ideas reflected the detailed feedback acquired from co-researchers about how to improve the wireframe, with a summary shown in Table 4.

**Table 4:** Suggestions to improve wireframe from co-researchers attending Workshop 2 for development of the *Sun Safe* app.

**Feature**	**Suggestion**	**Response^a^**
Home page	• Ability to use app without Internet access	• Some features accessible without Internet (Applied sunscreen, Notifications, Learn)
Notifications	• Sound specific to app	• A sound included for notifications
Weather data	• No comments made and little evidence this feature was accessed	• Make this feature more obvious on home page
Learn	• Additional content across all subtopics within Learn	• Web links to Cancer Council Australia^b^ and World Health Organization^c^ content
	• More pictures and color	• Development of app-specific artwork
	• External customizable “mini-games” (e.g., crosswords, puzzles)	• Puzzle generated of app icon artwork *via Zigsaw Planet* online platform^d^
	• Video content of a first-person account of a young person's experience with skin cancer	• Development of video content with Cancer Council WA—“Amelia's Story”
	• Links to websites to purchase sunscreens and related products	• Not considered appropriate for inclusion because of parental concerns
	• Capacity to contact an expert for more information	• Not considered a high priority
	• Capacity to donate to research	• Not considered a high priority
Applied sunscreen	• Needs to be adjustable	• Made adjustable (with 15-min time increments)
Quizzes	• Increase gamification elements such as having a leaderboard, timed sessions, vary question order	• Identified the *Quizziz* platform to host and develop quizzes, which has these gamification elements
	• Provide more colors and pictures	• *Quizziz* platform is visually appealing and images can be uploaded
	• Provide answers	• *Quizziz* provides answers
	• Lengthen quiz	• Quiz length increased to 20 questions
	• A badge/sticker for 100% correct	• Not possible due to budget constraints

a *Researchers assessed each suggestion, considering budget and other constraints, with a response to each suggestion listed*.

b *New content provided by links to websites of Cancer Council Australia websites (with permission): https://www.cancer.org.au/preventing-cancer/sun-protection/about-skin-cancer.html; https://www.cancer.org.au/preventing-cancer/sun-protection/about-sunscreen.html; https://www.cancer.org.au/preventing-cancer/sun-protection/vitamin-d/; and https://www.cancer.org.au/preventing-cancer/sun-protection/uv-alert/; https://www.cancer.org.au/preventing-cancer/sun-protection/preventing-skin-cancer/*.

c *New content provided by links to websites of World Health Organization: https://www.who.int/uv/uv_and_health/en/ and https://www.who.int/uv/faq/uvhealtfac/en/*.

d *See https://www.jigsawplanet.com/?rc=play&pid=0cdbdb785e69&pieces=25*.

### Researcher Feedback on Wireframe

Throughout a 7-week period, feedback acquired from the research team was similar to that provided by co-researchers, especially about increasing gamification of the “Quizzes” feature (e.g., requests for a leaderboard, more questions; Table 4). One suggestion was to change the name of the “Applied sunscreen” feature to “Sunscreen Timer.” Some of the research team were supportive of a more definitive game, to differentiate the wireframe further from other already existing online tools (e.g., SunSmart app), such as an augmented reality game similar to PokemonGo; however, this was beyond the budget contraints of this project. Furthermore, these types of games were not suggested by co-researchers throughout the interviews and Workshop 1 or 2. Additional feedback from the research team was to provide weather data (both UV Index and temperature) specific to the location of the user. Other minor changes to education content (in “Learn” and “Quiz”) were made based on recent updates in academic knowledge.

### App Development

A fully functioning app was developed in response to feedback of the wireframe obtained from co-researchers in Workshop 2 (Table 4) and the research team. The name of the app was not changed, as preferred names from Workshop 2 (Figure 5A) did not adequately describe the nature of the app (i.e., The Hot App) or were identical to already existing apps (i.e., Sun Smart). Other changes are described below.

#### Content Changes

In response to requests from participants attending Workshop 2 for more content in “Learn,” a series of weekly “push” notifications (Fun-SUN-facts) were added into the app as an additional means of providing education content to end users (Table 5). This was also done to emphasize educational concepts about vitamin D that were less well-rated by some co-researchers at improving their knowledge (Figure 6A), with other facts added that could not be (easily) obtained by reading the “Learn” feature directly (e.g., Fun-SUN-fact #3). More content was also added to “Learn” with additional links (to Cancer Council Australia and World Health Organization websites) included across all topics for users to find further information as desired (Table 4). To respond to a request for customizable “mini-games,” a link was provided to a puzzle generated using the UV Index icon (Figure 5B) using the *Jigsaw Planet* platform (https://www.jigsawplanet.com) to produce a freely accessible mini-game. In response to initial considerations by researchers and suggestions by co-researchers (Table 4) about incorporating an impactful first-person account, a video link was developed in which a young person recounted their experiences with melanoma (“Amelia's Story”) and added as a web link to the “Learn” content. This was specifically developed by Cancer Council WA.

**Table 5:** Proactive “push notifications.”

•Fun-SUN-fact #1: Oily fish (like salmon and tuna) are a good dietary source of vitamin D!
•Fun-SUN-fact #2: Shade does not provide complete protection from UV radiation, which can be reflected from the ground and other surfaces
•Fun-SUN-fact #3: You can't make vitamin D while driving in the car (with the windows closed) as the glass and tinting block the UVB rays from reaching your skin
•Fun-SUN-fact #4: Western Australia ranks second in terms of rates of skin cancer among Australian states and territories
•Fun-SUN-fact #5: The UVB rays of sunlight are most risky for sunburn, but are also needed to make beneficial vitamin D in your skin
•Fun-SUN-fact #6: Vitamin D is important for healthy bones, brains, and lungs

#### Modifications to “Quizzes” Feature

A Web-based environment scan was done searching for available online platforms that had gamification elements requested by co-researchers (Table 4). The *Quizziz* platform (quizziz.com) was selected in preference to other freely accessible education-based online quiz platforms because this platform:

- could be integrated into the app and used on iOS devices;
- is used by teachers in school settings in WA;
- is freely accessible with no ads;
- had flexible formatting of questions (e.g., single or multiple correct answers are possible);
- facilitated rapid modification of developed quizzes, with content password-protected and controlled by the quiz developer;
- provided correct answers following each question and at the end of the quiz;
- had high visual appeal with the capacity to upload images and branding (e.g., app icons and artwork);
- had gamification elements including a leaderboard, progress bar, optional music and sound effects, languages, funny memes and themes, timer, “power-up” settings, and built-in education tools (“shuffle cards”); and
- addressed parental concerns, with ongoing automated moderation filters that search for (and remove) inappropriate content.

A 20-question quiz was developed on *Quizziz* (also incorporating Fun-SUN-Facts of Table 5) and linked into the *Sun Safe* app, with users directed to bypass a “Google login/create account” by clicking on the “skip for now” link. Other possible platforms considered to host the quiz included *ClassMaker, OpinionStage, SurveyMonkey, Qualtrics* (UWA), *Playbuzz*, and *OpenTrivia*. However, none of these had as many advantages as listed above for *Quizziz* and were often limited by costs to access, a general lack of gamification, and/or the inclusion of ads. The name of the “Quizzes” feature was also simplified to “Quiz”.

#### Location-Based Weather Data

To provide location-based weather data, the *Dark Sky* application programming interface (API) (see https://darksky.net/dev) was sourced. This API allows geolocation-based weather data (worldwide) to be accessed for free for the first 1,000 downloads/day, with UV Index data calibrated to terrestrial ground conditions (including consideration of cloud cover; see also https://darksky.net/dev/docs/sources). Users of *Sun Safe* were requested permission when first downloading the app to share their location and IP address with the *DarkSky* API to obtain weather data at their particular location. No other user data could be sent to the *Dark Sky* API with all cross-origin resource sharing disabled. On the home page, that day's maximal temperature and UV Index were featured and an estimated time (to the hour) that these events would occur. The current temperature and UV Index were also shown in small font, as well as an icon to indicate the main weather condition for that day (e.g., a sun if sunny). As there was limited feedback from co-researchers on weather data (Table 4), the link to this information was made more obvious through a new button (“View this week”). It is important to note that beyond the end of 2021, alternate sources of weather data may need to be sourced so that this feature can be retained in *Sun Safe*, with *DarkSky* purchased recently by Apple (see https://blog.darksky.net, July 1, 2020).

#### Development of App-Specific Artwork and Notification Sound

A total of 35 designs were submitted by 6 artists to the 99designs platform, of which six were short-listed to be evaluated *via* an online poll. The final artwork used in the app (Figure 5B) received a mean of 4.3-star rating (from 9 voters) and was the highest rated. Comments on this artwork included “The images themselves are all very bright and eye-catching” and “Clearest design. Fastest to communicate.” These icons were also installed as a pack of “stickers,” which could be used when text messaging. The “piano arp down” sound (106812780) was selected by researchers from a choice of seven sounds as a non-intrusive but distinct notification sound to remind users to check the UV Index.

### Beta Testing of App

All issues or suggestions aiming to improve the app identified during beta testing are listed in Supplementary Table 5 and were considered for inclusion in the app. Changes were made that were possible within the budget constraints of this project. Firstly, a brief onboarding process was added for new users, which included describing how weather data (i.e., temperature and UV Index) were presented by the app, and important features of the app (i.e., sunscreen timer and daily notifications to check the UV Index). Additional movie content (*SunSmart tips from the Bondi Rescue Boys*, developed by Cancer Council WA) was identified and included as a web link in “Learn” (see Supplementary Table 5). Information from web links provided through the World Health Organization was redeveloped into infographics and more user-friendly formatting (developed by Telethon Kids Institute, Supplementary Table 5). Consumer research buddy feedback of the app from a meeting (May 31, 2019) held during this time included a suggestion to have multiple quizzes hosted within the “Quiz” feature of the app, such as an “easy” (10-question) quiz and a “hard” (20-question) quiz. These were both developed on *Quizziz* and incorporated into this feature. Minor issues about coding the geolocation-specific weather data and the day's date were identified and corrected during this time. All suggestions identified as priorities and/or were possible to modify within project constraints and budget (Supplementary Table 5) were made prior to Workshop 3, with the exception of onboarding and new internally hosted websites, so that further feedback on whether these changes were needed could be reviewed by co-researchers.

### Workshop 3 With Co-researchers: Testing the App

Co-researchers assessed the *Sun Safe* app completing the same qualitative worksheet as used for the wireframe in Workshop 2. Most co-researchers downloaded the beta version of *Sun Safe* onto an iPhone (10/15, 67%) or iPad (3/15, 20%) device (two did not state what device they used). Major themes that emerged from group discussions of the tested app were that most attendees liked the redeveloped “Quiz,” “Sunscreen Timer,” “View this Week,” and “Learn” features. There was also positive feedback on the new app-specific icons (artwork). Critical feedback included that some co-researchers preferred that the “Quiz” platform be built into the app (rather than hosted externally), the external information websites in “Learn” (especially from the World Health Organization) were boring, and some co-researchers disliked repetition of questions across the “Easy” and “Hard” quiz options. Some co-researchers also wanted the capacity to predict the UV Index across the day using the graphs in the “View this week” feature. These and further issues and suggestions raised by co-researchers during the qualitative assessment of the app are summarized in Table 6.

**Table 6:** Issues arising with *Sun Safe* app by co-researchers at Workshop 3.

**Feature tested**	**Issue or suggestion to improve app**	**Response^a^**
Notifications	•Capacity to write own notifications	•Not considered a priority
	•Ability to set notifications at different times each day	•Modifications made so that different times could be set for weekdays and weekend days
	•Capacity to set notifications multiple times a day	•Not considered a priority
	•Capacity to modify the way to receive notifications	•Not considered a priority
	•More colors	•Not considered a priority
	•Increase size	•Not possible within constraints of other features
View this week	•Current UV Index missing	•Change made
	•Only maximum temperatures shown	•Minimum temperatures not added as temperature is not the focus of app
	•Add legends to graphs	•Changes made
	•Highlight when sun protection is needed	•Highlighted in a box
	•Increase font size	•Change made
	•“Onboarding” needed	•Change made
	•Capacity to predict the UV Index across the day using the graphs	•Not done as significant coding required to complete this suggestion
	•Detailed weather information is missing	•Not added as it is not the focus of app
	•Add scale to graphs	•Not done as significant coding needed
Learn	•A few co-researchers wanted more information, while others wanted less	•Most co-researchers are happy with the quantity of information presented
	•More colors, pictures, and images	•Not considered possible as each section has an icon, followed by information in dot-points, sometimes another image/graphic and 1–3 web links
	•Develop internally hosted web pages for more user-friendly content with infographics^b^	•New internally hosted websites developed with infographics
	•More information on what to use and when to apply sunscreen	•Not considered necessary as 2 web links provided
Sunscreen timer	•Merge with notifications	•Not possible as would require rebuild of app
	•Capacity to set more times	•Not considered a priority
	•“Onboarding” needed^b^	•Change made
Quiz	•Build and embed into app	•Not possible to retain gamification elements provided by *Quizziz* platform with current budget
	•Remove duplicated questions from the “Easy” and “Hard” quizzes	•Change made
	•More fun questions	•Change made
	•Add prize (e.g. “coins,” avatar)	•Not possible with budget constraints

a *Researchers assessed each suggestion, considering budget and other constraints, with a response to each suggestion listed*.

b *Also raised during beta testing (see Supplementary Table 5) but not modified until after Workshop 3*.

The semiquantitative evaluation of the of *Sun Safe* app (Figure 6B; completed by 14/15 participants) was very similar to those given for the wireframe (Figure 6A) across all seven items (*p* ≥ 0.23, Mann–Whitney tests). Co-researchers rated the “Learn” content the highest compared to the real-time weather and UV information, “Quiz,” and “Sunscreen Timer” (Figure 6C; data obtained from 13/15 participants), although there was no significant difference in how these features were rated (*p* = 0.56, Kruskal–Wallis test). Push notifications (Table 5) could not be assessed by co-researchers during the short time frame of this workshop, with feedback on the “Notifications” feature not requested for the same reason. By a show of hands, most co-researchers indicated that they liked the “stickers” of artwork/icons that could be used when text messaging. Researchers again assessed each idea or suggestion raised by co-researchers for feasibility for inclusion in the final version of the app to be pilot tested (Table 6), with all suggestions identified as priorities and/or within budget constraints made prior to review for governance approvals to test the developed app in pilot intervention studies within a local school at a later date.

### Further Modifications to Enable Pilot Testing

**Figure 7:**
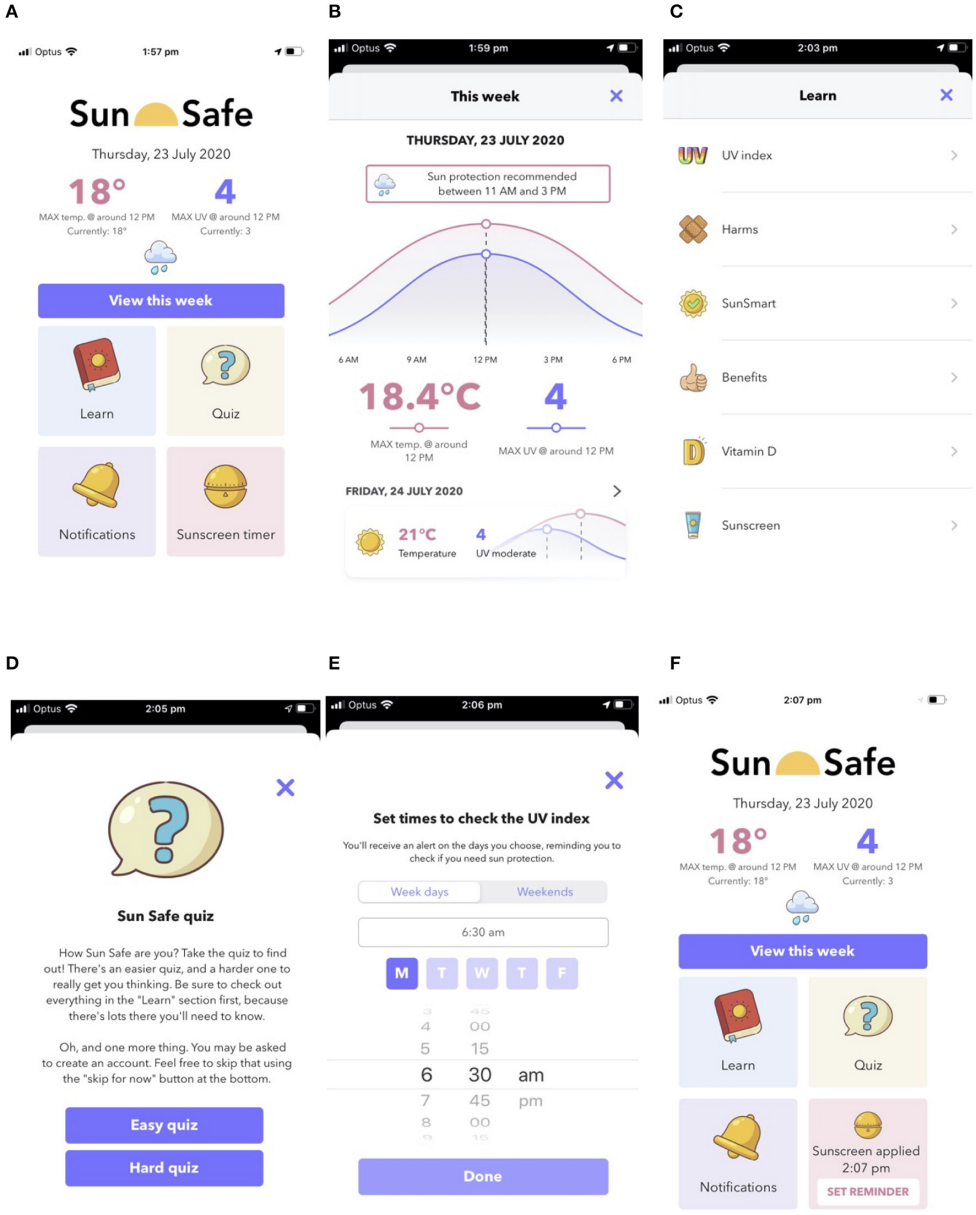
Screenshots of the fully functional Sun Safe app to be tested in intervention studies. Features of the app included: **(A)** home page with that day's maximal temperature and UV Index and access to other features; **(B)** more detailed predictive data on the maximal temperature and UV Index linked to times when to use sun protection for that day and subsequent days, accessed through “View this week” button; **(C)** educational content in “Learn” feature; **(D)** “Easy” and “Hard” quizzes in “Quiz” feature; **(E)** capacity to set daily “Notifications” to check the UV Index; and **(F)** capacity to set a “Sunscreen Reminder” (through “SET REMINDER” button) for up to 2 h from when sunscreen is first applied, with a notification sent to reapply.

Minor changes to the *Sun Safe* app were made as part of the governance approval process of the Department of Education of Western Australia. An important concern raised by reviewers as part of this process regarded data privacy, specifically whether there was any data collection from app users when using the *Sun Safe* app. No data were collected *via* the *Sun Safe* app directly, with the *Quizziz* platform accessed without creating a user account, although the *DarkSky* API required the location and user's IP address data to provide localized weather data. Other issues raised during the governance approval process were the web links to information provided by World Health Organization, with concerns raised about the possibility of young users accessing sensitive or inappropriate information. This issue was resolved by providing this information on internally hosted websites at the Telethon Kids Institute. Finally, the *Jigsaw Planet* online platform (Table 4, Supplementary Table 5) was considered a possible risk for young users, and in response to this concern, web links to these puzzles were deleted. The version of the *Sun Safe* app to be pilot tested for effectiveness (performance under real-world conditions) in changing sun health knowledge and behavioral change is depicted in Figure 7.

### Future Planned Pilot Intervention Studies

Future pilot studies will test the feasibility of determining the effectiveness of the *Sun Safe* app in improving sun health knowledge and behaviors by young adolescents. Feasibility outcomes will include determining how to best recruit young teenagers into intervention studies and comparing community-based social media strategy with a school-based workshop approach. We will also develop and assess new tools to measure sun health knowledge (e.g., multiple-choice test), personal sun exposure, and skin health [*via* questionnaire and dosimetry (31, 32)] and assess the quality of the developed app [modified uMARS survey (33)]. The best means to acquire data using these tools will be evaluated, comparing online with more traditional (i.e., paper-based) methods. We hypothesize that a combined approach may be needed to effectively recruit participants and assess the effectiveness of *Sun Safe* in which the app is downloaded and used without further encouragement. School-based approaches may be suitable, with the World Health Organization recognizing schools as effective settings for skin cancer prevention efforts (34). Indeed, other sun health apps have been assessed in school settings with successful recruitment, although limited by some loss to follow-up (19).

Development of apps to use as learning tools in secondary education may be appropriate to build on earlier education efforts around sun safe behaviors in Australia. A national parliamentary inquiry into skin cancer in 2015 highlighted a lack of progress in embedding healthy sun behaviors in young Australians at secondary schools and recommended expanding high school curricula to cover healthy sun-aware behaviors (35). Koch et al. (9) also identified that “*new approaches are needed to minimize reactance responses in adolescents while fostering favorable attitudes to sun protection*.” A previous lack of progress may be in part explained by the expected growing maturity and self-reliance of adolescents, their resistance to parental influence and targeted public health messages, and increased risky behaviors around sun exposure [reviewed in Koch et al. (9)]. The freely accessible *Sun Safe* app was well-rated by most co-researchers, with most giving subjective ratings of 4 or more when asked if it was easy to find information (12 of 14) using the app and if the app helped them better understand the UV Index (12 of 14) (Figure 6B). The *Sun Safe* app could thus be a complementary education tool to use in secondary school settings, particularly in WA, as the Health and Physical Education Curriculum includes *Sun Protective Behaviors* as a learning outcome in Year 7, which is delivered to 12- and 13-year-olds (36).

### Comparing *Sun Safe* With Other Sun Health Smartphone Apps for Young People

In addition to *Sun Safe*, other smartphone apps are available that provide information on the UV Index [e.g., *SunSmart* app, Cancer Council Australia (37); *Sunwise UV Index*, Environmental Protection Authority, USA]. However, these apps target interested and health-conscious adult populations, and few are designed specifically for adolescents. The *Mollie's Fund* app aims to improve the skin cancer knowledge of children and adolescents. Others, such as *Cache-cache Soleil* (French League Against Cancer), provide only minimal education about sun safety and are likely only appealing to very young children. More relevant apps include the *SunFace* app, which asks users to take a photo of their face, which is then aged with and without sun protection (20). This app has been tested in secondary and tertiary education settings and may reduce sun-tanning behaviors (19, 20) and increase sunscreen use by young participants (19). Ongoing challenges in developing health promotion tools for sun health, especially in the online intervention space, include engagement of male participants (19) and determining how to best design placebo control groups.

### Limitations

This study was limited by recruitment of co-researchers from a single location (Perth, WA), with some uncertainty as to how the findings here are applicable elsewhere. There was also gender imbalance in recruitment, with more females than males, although fewer male co-researchers were lost to follow-up through participation in successive workshops (Figure 1). Some male co-researchers were observed to be “turned off” or “quiet” during workshops. Therefore, there is uncertainty as to how feedback from male co-researchers translated into the development of the *Sun Safe* app. Other limitations include potential “social desirability bias” in responses, as even though all surveys were anonymous, co-researchers may have tried to present themselves favorably by answering questions more positively to satisfy researchers (or their peers), potentially reducing the validity of their responses (38). Other limitations included that we did not collect demographic data related to ethnicity and skin type, which could impact the way people spend time in the sun. Finally, recent studies suggest that using the UV Index to direct sun behaviors may have both positive impacts (e.g., increased shade use and wearing of long-sleeved clothing) and unintended consequences (e.g., increased tanning, reduced sunscreen use) (39). Therefore, assessment of the effectiveness of *Sun Safe* in modulating sun health knowledge and behaviors of the target group (i.e., young adolescents) is necessary.

## Conclusions

We developed *Sun Safe*, an iOS app that aims to improve health knowledge and behaviors of young adolescents regarding safe sun exposure. Through a highly consumer-informed process, we produced an app with features including real-time weather information on the UV Index (“View this week”), reminders to check the UV Index (“Notifications”) and reapply sunscreen (“Sunscreen timer”), educational content (“Learn”), and gamification strategies (“Quiz”). The *Sun Safe* app was well-rated by co-researchers involved in its development and is now being tested for its capacity to improve the knowledge and sun practices of young adolescents.

## Data Availability Statement

The original contributions presented in the study are included in the article/Supplementary Material, further inquiries can be directed to the corresponding author/s.

## Ethics Statement

The studies involving human participants were reviewed and approved by Human Research Ethics Committee of the University of Western Australia (RA/4/20/4424). Written informed consent to participate in this study was provided by the participants' legal guardian/next of kin.

## Author Contributions

SG, RN, GA, LB, PH, RL, and MS contributed to the conceptualization, writing, review, and editing. SG and RN contributed to the methodology. SG contributed to the formal analysis, writing the original draft, supervision, project administration, and funding acquisition. IC and NG contributed to the data curation. MJ contributed to the wireframe. JW contributed to the app development. All authors have read and agreed to the published version of the manuscript.

## Conflict of Interest

MJ was employed by Curve Tomorrow. JW was employed by Reach Health Promotion Innovations. The remaining authors declare that the research was conducted in the absence of any commercial or financial relationships that could be construed as a potential conflict of interest.
