# Supplementary material for: Developing an Online Tool to Promote Safe Sun Behaviors With Young Teenagers as Co-researchers
Source: Front Digit Health. 2021 Mar 23;3:626606. doi: 10.3389/fdgth.2021.626606 (PMC8521794; doi:10.3389/fdgth.2021.626606)
Supplement: Supplementary file 1 [file Data_Sheet_1.PDF]

Supplementary Material

Methods Article: Nguyen et al. *Developing an online tool to promote safe sun behaviours with young teenagers as co-researchers.*

Supplementary Methods

2.1 Ethics and governance

All research staff and students interacting with young people < 18 years of age obtained *Working with Children Checks* (State Government of Western Australia). Approval was obtained from the Education Department (State Government of Western Australia) by submitting an *Application Form for External Parties to Conduct Research on Department of Education Site* to conduct a pilot intervention of the online tool in a local school.

2.4 Participant ('co-researcher') recruitment

Participants received an honorarium of $25 for participating in telephone interviews, and $20 per hour (AUD) for time spent participating in workshop activities, and a 2-hour parking voucher for each workshop. The value of these honoraria was determined based upon advice from consumer advocates at the Consumer and Community Health Research Network (WA).

2.5 Consumer recruitment

Research buddies can be "consumers and community members that provide links between the researcher, consumers and community members, organizations and/or research funder" (1). Independent consumer advice was considered necessary as the young people directly involved in the research project as co-researchers would likely have some conflict of interest brought about through their investment and participation in the research process and developed online tool, which might have altered their capacity to assess the progress of the project in an unbiased fashion. Consumer research buddies were recruited via the Involvement Network of the Consumer and Community Health Research Network and received an honorarium of $30 per hour (AUD), and a 2-hour parking voucher for their time spent attending meetings (4 meetings were held in April and June 2018, and May and June 2019) and reviewing documents.

2.6 Design Thinking for online tool development

The Design Thinking approach prioritizes the needs and challenges of end-users, with the goal of developing more effective solutions (2). With ongoing input from end-users, those knowledgeable about the health problem and technologies, different options were considered to achieve the desired results, allowing also for budget and time constraints for the rapid development of a fully-functional app for further evaluation. It was hypothesized that implementing Design Thinking theory and processes, and incorporating content, plans, format, strategies and language ideas from all stakeholders, would facilitate the development of an online tool that would be highly likely to meet the needs of end-users and all stakeholders.

2.7 Interviews of co-researchers: initial information gathering

Interviews were used in addition to workshops, so that information could be gathered outside a group setting and avoid the bias that sometimes occurs in group situations, where the opinions of some dominate and result in the loss of creativity and independent thinking (i.e. 'group-think'). We recruited 10 individuals for the interviews, as prior experience suggested that at least 5 individuals are needed to generate sufficient knowledge for the development of wireframe (independent to knowledge gained through Workshop 1) for further testing.

2.9 Workshop 1 with co-researchers: brainstorming to develop an online tool

A light meal was provided to participants during each workshop, which were all held in a seminar room at Telethon Kids Institute (level 6, Perth Children's Hospital, Nedlands, WA). Up to 20 individuals participated in workshops to gather sufficient input, and varied responses from male and female participants from the community. In workshop 1, participants (co-researchers) were seated at 1 of 4 tables (5 individuals/table). Co-researchers were first briefly introduced the aims of the research project and provided with the definition of the UV Index. An 'ice-breaker' was then conducted to allow participants to get to know others at their table, and as a larger group. For each discussion topic, table facilitators summarized the two main points that arose from their table to the wider group, which were recorded.

2.11 Workshop 2 with co-researchers: testing the wireframe

Co-researchers were asked to bring or were loaned an iOS device (iPhone or iPad) to test the wireframe. Co-researchers were allowed to sit where they wished on 1 of 3 tables, with 6 individuals/table, with an initial 'ice-breaker' conducted. For each feature of the wireframe, co-researchers were asked to record why that feature was selected, what they liked and disliked (and why), and provide suggestions to improve the selected feature. Extra space was provided for co-researchers to record any further comments.

2.15 Workshop 3 with co-researchers: testing the app

Participants were asked to bring their own iOS iPhone or iPad, with devices provided to those who did not bring their own. Prior to testing the app, an initial 'ice-breaker' was conducted. Co-researchers were given time to discuss their opinions of the app as a whole group.

Supplementary Results

3.1 Requirements, constraints and considerations of online tool

A theory utilized in the development of the online tool included the Health Belief Model, which explains and predicts health behaviours focusing on attitudes and beliefs of the individuals involved. In this model, a person will undertake a health-related action if they feel they can avoid a negative health condition (e.g. skin cancer or vitamin D deficiency), has a positive expectation that by undertaking the action they will avoid the health condition, and that they can successfully undertake the health action (3). A second related theory was Social Cognitive Theory, which focuses on how behaviours of young adolescents are informed by observational learning, expectations and self-efficacy, and, identifies the impact of environmental influences where individuals learn from each other

via observation, imitation and modelling (4). Finally, Self-Determination Theory, in which young people are empowered to act in healthy ways by a supportive environment through feeling connected as part of a group, was an important theory used as part of development of this tool (5).

Supplementary References

1. McKenzie A. Consumer and Community Participation Fact Sheet M07: Research Buddies (Consumer and Community Health Research Network (Western Australia)) 2011.

2. Roberts JP, Fisher TR, Trowbridge MJ, Bent C. A design thinking framework for healthcare management and innovation. Healthc (Amst). 2016;4(1):11-4.

3. Janz NK, Becker MH. The Health Belief Model: a decade later. Health Educ Q. 1984;11(1):1-47.

4. Bandura A. Social Learning Theory. Englewood Cliffs: Prentice Hall. 1977.

5. Sheldon KM. The self-determination theory perspective on positive mental health across cultures. World Psychiatry. 2012;11(2):101-2.

Supplementary Tables

STable 1. Prompter questions to help with table discussion during Workshop 1.

**1. Sun protection and harms – When you spend time outdoors with friends and family, how do get ready to be sun safe?**
• Think about the last time you went out on a sunny day. How did you prepare yourself?
• Have you been sunburnt? Why? How would you prevent this next time?
• Has someone you know had skin cancer?
• How do you find what the UV Index or weather is going to be?
• Imagine this. You know you get sunburnt easily. You are going to out with your friends on a sunny day. You have a hat, but nobody is wearing one. Will you wear your hat? Why/Why not?
• What stops you using sun protection?
**2. Sun exposure benefits – What have you heard about the benefits of sun exposure?**
• Have you heard of vitamin D?
• What is vitamin D good for?
• Has someone you know had their blood levels of vitamin D tested?
• Do you know of any other benefits of sun exposure?
• How would you like to learn about vitamin D/UV Index?
**3. 'How-might-we' brain-storm[1] – How might we use technology to support sun safe practices?**
• Your phone
• The internet
• What apps do you use? What features of these do you like? How might you incorporate sun safe practices (think about activities 1 and 2 of this workshop)?
• How do you prepare for outdoor activities?
• A wearable (e.g. Fitbit)

[1]Co-researchers were instructed that all ideas were useful contributions, and encouraged to 'think outside the box'

STable 2. Themes identified in Interviews.

Major themes[1]	Content sub-themes
1. Major strategies	• Seek help from parents (usually from Mum)<br>• Look up weather (usually on smartphone app)<br>• Look outside<br>• Use current season to decide<br>• Follow school rules, teacher's instructions
2. Challenges	• Don't understand UV Index in different weather<br>• Don't like wearing hats all the time<br>• Conflicting information about sun protection<br>• Secondary school policies are inconsistent<br>• Current online tools rarely provide data on local UV Index<br>• Boys don't care, and don't think about sun health
3. Emotions	• Don't want to be different from peers (i.e. wear a hat)<br>• Would rather have school enforce rules so they aren't different (from their peers)<br>• Have been burnt before, was painful, and so don't want to experience that again<br>• Getting a slight burn on nose or cheek isn't that serious<br>• I'm a bit tanned so I won't get burnt<br>• Sun exposure can make you have bad skin<br>• More serious about sun protection if they know someone who has skin cancer<br>• Worried about certain types of moles<br>• Decisions are made rapidly (in less than 5 seconds)<br>• Anxiety around getting burnt
4. Needs	• Information about UV Index<br>• Being notified about when to reapply sunscreen<br>• See the results of not being SunSmart<br>• Information around different sunscreens<br>• Explaining the benefits of vitamin D<br>• More fashionable hats<br>• Make-up (cosmetics) with sun protection factor (SPF)

[1] Identified through rapid assessment of audio-content of interviews of co-researchers

STable 3. Specific responses from co-researchers allocated into major themes which emerged during the ‘How might we’ brain-storming activity[1] of Workshop 1

Theme 1: **Methods on how to promote safe sun practices**	***Theme 2*:** **Means to remind and notify individuals**	***Theme 3*:** **Information on how to effectively use sunscreens**	***Theme 4*:** **Games and quizzes**
A play about how important vitamin D is[2]	Notification of weather	App that shows (through a UV lens) where you have put sunscreen	Games about skin cancer facts
A show that gives us more information about vitamin D	We could make a UV rate app on all phones when you buy it, you get a notification every day so you don’t even need to open the app. Make it so you can’t turn off the notifications or delete.	Able to scan your body to tell you where sunscreen is so if you have a spot you know	Design a game or build on a popular game platform, saying that you can earn more in-game money or clothes for your avatar if you put sunscreen on your avatar or identify skin cancers
Skits/videos that convey information in a fun way	Weather app that advises when to wear a hat and sunscreen depending on the weather	Show colours of skin and how long it will usually take to get sunburnt without sunscreen	A game of some sort
Interactive learning app for vitamin D	Be able to search time of day and show UV at that time	Special sunscreens for different body colours	Fun quizzes
A chart of the weather so the younger kids can see the weather and how much sunscreen they need to put on	A reminder in the morning (or whenever you need) to apply sunscreen	A camera that shows you the UV rays what type of sunscreen you need and you put your skin colour and it would tell	Fun quizzes about sunsmart and vitamin D and UV rays
TV commercials/insta<br>Famous people promoting sun safe	A weather type app but it tells you what the UV Index is	Online ‘shopping’ (including) sunscreen foundation with SPF incorporated, hats, clothing	You could create games to make kids realise that sun be good and bad
Get celebrities to say the information so people will be more engaged	Instagram story or snapchat story (a sunsafety reminder) to remind teenagers about sun safety	What sunscreens have an affect and what doesn’t	A whole playground with a maze, and in the maze at every dead end, there is a fact, like 1 in 4 people were sunscreen. And before the end, there is a secret door, which will only let you pass to the end if you answer a question correctly, like “what do you need to before going out in the sun?”
Cartoon visual information	Reminders to apply sunscreen and sun protection	Waterproof sunscreen	Fast response game<br>Battle-games Royale
Method to handle bad sunburns	Notification of when to reapply sunscreen	A phone box-like place at the beach that you pay $2-3 (or over app on phone) and you go inside and it sprays you with sunscreen inside	App that gives info on sun safety with a short quiz afterwards to see how much your remembered

Sponsor a football team	A reminder app that tells you when to put on sunscreen and what types of sunscreen		A board game or video game or anything like that to teach us
Methods on how to handle sunburn freak outs	Reminders when you have to apply sunscreen		
Skin patch that changed in a picture once enough sun	Watches with notifications when you need to apply sunscreen		
People's experience with the sun in a video (good and bad experiences)	Wearable tech: watchband that changes colour		
Use cartoons and bright colours	UV Index for whole week		
Fun random facts that you will remember	When the highest UV sun ray is in the day – notify you when it is		
Parody of a pop song with sunscreen facts			
Netflix movie			
Someone walking along population beaches reminding people to wear sunscreen and offering free sunscreen and hats			
Cartoons on to for kids and teenagers about sun safety			
Animated pictures/GIFs of UV info			
Filter/add on for the UV Index on the camera app of your phone			
Activity-specific app set for kayaking, running, sport.<br>Royal show app			
Punishes you with a zap or embarrassing noise			
Solar-powered patch that zaps you when you've had enough sun			
Sun exposure meter built into cycle computer			
Program a section for children, teenagers and adults			
You … all the info you need to know			
Invention: conditioner and shampoo with SPF in it			
Fitbit just for sun stuff			
PSA[3] type things online			
uTube adverts			

Use quick advertisements to briefly inform them of the dangers of the sun			
App with daily advice that gives points that can be exchanged for vouchers			
Write it on the sky			
TED talks			
Famous people promote sun safety			
Find a catch phrase about skin cancer			
Make advertisements for the apple weather app (everyone should get apply devices)			
Apps for schools to use (primary schools) and high schools even			
Find a cheese mascot			
Parodies of pop songs with sun safe info			
You could use a uTube video			
A podcast			
Instagram account to help teenagers understand about sun safety and how important it is to be protected, in a cool way			
Instagram. Use new SunSmart account to raise awareness of skin cancer and sunburn			
Wear a hat and sunscreen<br>Explain what vitamin D is with a video			

[1]The How-might-we topic was, "How might we use technology to support safe sun practices?" with co-researchers (*n*=20) asked to rapidly (in a 25-min session) and confidentially record their ideas on post-it notes which were then themed by researchers into 4 major areas.
[2]Recorded verbatim (as written on post-it by co-researcher)
[3]PSA = public service announcements

STable 4. Specific responses from co-researchers allocated into major themes which emerged during the How-might-we brainstorm activity[1] of Workshop 2

***Theme 1:* 'Mini'-games & Ways to Improve 'Quiz'**	***Theme 2*: Avatars & BitMoji rewards**[2]	***Theme 3*: Music**	***Theme 4*: Vouchers**	***Theme 5*: Miscellaneous**
Crossword<br>Hangman<br>Wordsearch [3]	Avatars = points you collect from sunsafe games can go towards new avatars, pets for avatars and clothing	Songs, themes songs about suns safety in a karaoke style	Gift vouchers<br>• City Beach[4]<br>• Kmart[4]<br>• Cotton On[4]<br>• Supre[4]<br>• Transit[4]	Cartoons for SunSmart<br>(Short movies)
You can connect to friends and play a game with them	BitMoji/personal avatar	Theme song	Vouchers<br>• Typo[4]<br>• Smiggle[4]<br>• I-tunes<br>• Boost[4]	Online donations to those who have cancer
Cooking games<br>Stories (snapchat)<br>Puzzles<br>Merch<br>Point systems (for ads)	BitMoji	Really annoying music that gets stuck in your head	Where to shop<br>• Priceline[4]<br>• Chemist Warehouse[4]<br>• Myer[4]<br>• Kmart[4]<br>• Target[4]	Comment place where you can ask questions
Leaderboard	Credits that you earn from quizzes to dress up your avatar		Voucher<br>• McDonalds[4]<br>• Priceline[4]	Could it be a website also for computers (I'm not aloud my phone during school) also for people who do not have a phone
Limit time to finish quiz	Customize your bitMoji		Vouchers<br>• Priceline[4]<br>• Myers[4]<br>• Maccas[5]<br>• EB games[4]	Asks you question about your skin colour, how active you are (what sport you are doing) to show you the most recommended sunscreen level
Points for correct answers			Eb-games[4] (x10) giftcards	Search a time and it shows an estimate for weather and UV
Hangman<br>Guess the cell (cancer)				Phone wallpapers
Different game options like games similar to pacman, crossy roads or subway surfer but including things about the sun				Online shopping<br>• Foundation with SPF<br>• Clothing<br>• Hats<br>• Sunsafe lipbalm

Sorting game				App merch
Puzzles				
Recipe game				
Games get a given a clue and you have to guess the word, every correct word gives you more points				

[1] The How-might-we topic was, "How might we use games, increase fun and engagement of the developed wireframe?" with co-researchers (*n*=19) asked to rapidly (in a 25-min session) and confidentially record their ideas on post-it notes which were then themed by researchers into 4 major areas.
[2] Bitmoji = personalised emoji, cartoon avatar
[3] Recorded verbatim (as written on post-it by co-researcher)
[4] Refers to specific Australian vendors
[5] Colloquial (Australian) slang for McDonalds Restaurants

STable 5. Issues arising with *Sun Safe* app during beta-testing

Feature tested	Issue or suggestion to improve app	Response[1]
General	• 'On-boarding' needed to introduce main features of the app	• Brief on-boarding process added after initial use of app
Homepage	• Include details of current location[1]<br>• Include an image for today's weather	• Not possible with budget constraints<br>• Added to homepage
Notifications	• On-boarding needed<br>• Minor wording changes suggested	• On-boarding added for this feature<br>• Changes made
View this week	• Include a degree sign for temperature	• Degree sign added
Learn	• Font size and minor content and word corrections suggested<br>• Include a new weblink[2] to content developed by Cancer Council NSW<br>• Add word captions into "Amelia's Story" video for those with hearing difficulties<br>• Include image of 'Slip, Slop, Slap, Seek, Slide' messaging developed by Australian Cancer Councils<br>• Add video developed by Australian Cancer Councils describing how to apply sunscreen<br>• Include a 'puzzle' for each learn feature[3]<br>• Develop internally hosted webpages for more user-friendly content with infographics[4]<br>• Add more video content	• Changes made<br>• Added in; however, this website was closed down during beta-testing<br>• Word captions added to video content<br>• Image included (with permission for use obtained)<br>• Video included (with permission for use obtained)<br>• Added in; however, these were later removed due to privacy concerns<br>• New internally hosted websites to be developed with infographics<br>• More video content added[5]
Sunscreen Timer	• Increase time selections to 15 min increments until 2 hours	• Change made
Quiz	• Include multiple quizzes<br>• Wording on images difficult to see<br>• Minor content and word corrections<br>• Add some 'fun questions' for teenagers<br>• Increase the time allocated to answer some questions in the 'Hard' quiz	• 'Easy' and 'hard' quizzes developed<br>• Font size increased<br>• Changes made<br>• Some 'fun' questions added<br>• Time allocations increased in length

[1] Researchers assessed each suggestion, considering budget and other constraints with a response to each suggestion listed
[2] http://www.yourtimeinthesun.com – n.b. not included in final tested app as this content was disabled
[3] Similar to that done for UV Index icon – see Table 4
[4] Using content provided by websites of World Health Organization (see Table 4), the following websites were developed by Telethon Kids Institute: https://www.telethonkids.org.au/be-involved/participate-in-research/sunsafe-study/factsheets/uv-and-health/; https://www.telethonkids.org.au/be-involved/participate-in-research/sunsafe-study/factsheets/health-benefits-of-sun-exposure/; and, https://www.telethonkids.org.au/be-involved/participate-in-research/sunsafe-study/factsheets/more-about-uv-harm-for-eyes/
[5] Using content developed (and with permission for use from) by Cancer Council WA: https://www.youtube.com/watch?v=6W0cCJQmt7w
